# Novel Fri1-like Viruses Infecting *Acinetobacter* *baumannii*—vB_AbaP_AS11 and vB_AbaP_AS12—Characterization, Comparative Genomic Analysis, and Host-Recognition Strategy

**DOI:** 10.3390/v9070188

**Published:** 2017-07-17

**Authors:** Anastasia V. Popova, Daria G. Lavysh, Evgeniy I. Klimuk, Mikhail V. Edelstein, Alexander G. Bogun, Mikhail M. Shneider, Artemiy E. Goncharov, Sergey V. Leonov, Konstantin V. Severinov

**Affiliations:** 1 Institute of Antimicrobial Chemotherapy, Smolensk State Medical University, Kirova 46а, Smolensk 214019, Russia; Mikhail.Edelstein@antibiotic.ru; 2 Moscow Institute of Physics and Technology (State University), Institutskiy per. 9, Dolgoprudny, Moscow Region 141700, Russia; sl@pharmcluster.ru; 3 State Researсh Center for Applied Microbiology and Biotechnology, Obolensk, Moscow Region 142279, Russia; bogun@obolensk.org; 4 Institute of Molecular Genetics, Russian Academy of Sciences, Kurchatov Square 2, Moscow 123182, and Institute of Gene Biology, Russian Academy of Sciences, Vavilova 34/5, Moscow 119334, Russia; lavyshd@gmail.com; 5 Skolkovo Institute of Science and Technology, Skolkovo 143026, Russia; E.Klimuk@skoltech.ru; 6 Shemyakin-Ovchinnikov Institute of Bioorganic Chemistry, Miklukho-Maklaya 16/10, Moscow 117997, Russia; mikhailshneider@gmail.com; 7 North-Western State Medical University named after I.I Mechnikov, Kirochnaya 41, Saint Petersburg 191015, Russia; 8 Institute of Experimental Medicine, Akademika Pavlova 12, Saint Petersburg 197376, Russia; Artemii.Goncharov@szgmu.ru; 9 Saint Petersburg State University, Universitetskaya emb. 7/9, Saint Petersburg 199034, Russia; 10 Peter the Great St. Petersburg Polytechnic University, Polytechnicheskaya 29, Saint Petersburg 195251, Russia; 11 Waksman Institute, Rutgers, the State University of New Jersey, Piscataway, NJ 08854, USA; severik@waksman.rutgers.edu

**Keywords:** bacteriophage, *Acinetobacter baumannii*, *Podoviridae*, *Fri1virus*, RNA polymerase, tail spike, capsule types

## Abstract

*Acinetobacter baumannii* is a gram-negative, non-fermenting aerobic bacterium which is often associated with hospital-acquired infections and known for its ability to develop resistance to antibiotics, form biofilms, and survive for long periods in hospital environments. In this study, we present two novel viruses, vB_AbaP_AS11 and vB_AbaP_AS12, specifically infecting and lysing distinct multidrug-resistant clinical *A. baumannii* strains with K19 and K27 capsular polysaccharide structures, respectively. Both phages demonstrate rapid adsorption, short latent periods, and high burst sizes in one-step growth experiments. The AS11 and AS12 linear double-stranded DNA genomes of 41,642 base pairs (bp) and 41,402 bp share 86% nucleotide sequence identity with the most variable regions falling in host receptor–recognition genes. These genes encode tail spikes possessing depolymerizing activities towards corresponding capsular polysaccharides which are the primary bacterial receptors. We described AS11 and AS12 genome organization and discuss the possible regulation of transcription. The overall genomic architecture and gene homology analyses showed that the phages are new representatives of the recently designated *Fri1virus* genus of the *Autographivirinae* subfamily within the *Podoviridae* family.

## 1. Introduction

The emergence and rapid spread of multidrug-resistant (MDR) and extensively drug-resistant (XDR) bacterial pathogens in medical practice necessitates the introduction of alternative methods of controlling antibiotic-resistant bacterial infections. This is especially pertinent for pathogens causing nosocomial infections, such as *Acinetobacter baumannii*, a gram-negative, non-fermenting aerobic bacterium. This microorganism has, over the years, acquired resistance to most currently available antibiotics, disinfectants and antiseptics, tolerance to detergents, ultraviolet radiation, desiccation, and the ability to form biofilms on various biotic and abiotic surfaces [1]. *A. baumannii* is often associated with nosocomial pneumonia, wound and catheter-related urinary tract infections, postsurgery complications, and bloodstream infections, especially in severely ill or immunocompromised patients in intensive care and burn units [1,2].

One of the potentially attractive alternatives to solve the problem of spread of antibiotic resistant strains is the use of lytic bacteriophages, as well as bacteriophage-derived antibacterial enzymes and proteins. Generally, lytic bacteriophages initiate the production of progeny virions by inhibiting or modifying vital cellular processes.

Over the last five years, a number of *A. baumannii* phages that are promising for practical use have been reported. Most of them are representatives of the *Myoviridae* [3,4,5] and *Podoviridae* [5,6,7,8] families. In this work, we present a characterization of two podoviruses, AS11 and AS12, novel members of the genus *Fri1virus* (named after the podophage Fri1) recently designated by the International Committee on Taxonomy of Viruses (ICTV) [9], and compare them to related phages. 

## 2. Materials and Methods 

### 2.1. Bacterial Strains and Their Identification and Characterization

Multidrug-resistant (MDR) and extensively drug-resistant (XDR) *A. baumannii* isolates (*n* = 100) were collected from various clinical specimens (blood, respiratory tract, intra-abdominal, urinary tract, skin and soft tissues, and spinal fluid) of hospitalized patients as part of the multicenter national surveillance studies on nosocomial infections in Russia and Belarus in 2002–2013 [10]. Non-clinical isolates (e.g., those obtained from screening samples of patients without signs of infection) were not allowed. The initial species identification of isolates was performed at local clinical microbiology laboratories, where they were isolated; final identification was carried out at the coordinating laboratory of the Institute of Antimicrobial Chemotherapy, Smolensk State Medical University, Smolensk, Russia, by means of MALDI-TOF mass spectrometry (MALDI Biotyper, Bruker, Germany), and by real-time PCR detection of *A. baumannii* species-specific *bla*_OXA-51-like_ beta-lactamase genes. All isolates were also tested for the presence of acquired carbapenemase genes of *bla*_OXA-23_, *bla*_OXA-24/40_, and *bla*_OXA-58_ groups by commercial real-time PCR (Amplisens MDR Ab-OXA-FL, Central Research Institute of Epidemiology, Moscow, Russia). Susceptibility testing of *A. baumannii* isolates to ceftazidime, cefepime, imipenem, meropenem, sulbactam, gentamicin, amikacin, ciprofloxacin, levofloxacin, colistin, tigecycline and trimethoprim-sulfamethoxazole was performed by the reference broth microdilution method according to ISO 20776-1:2006; the results were interpreted according to EUCAST clinical breakpoints, v. 6.0. Isolates insusceptible to at least one agent in three or more antimicrobial categories (e.g., carbapenems, fluoroquinolones and aminoglycosides) were considered MDR; isolates insusceptible to at least one agent in all but two or fewer categories were considered XDR.

The genetic diversity of *A. baumannii* isolates was evaluated by single nucleotide polymorphism (SNP)-typing using a set of 21 informative SNPs from 10 chromosomal loci (*glt*A, *rec*A, *cpn*60, *gyr*B, *gdh*B, *rpo*D, *fus*A, *pyr*G, *rpl*B, and *rpo*B) used in the two multilocus sequencing typing (MLST) schemes by the University of Oxford, Oxford, UK (Oxf) [11] and the Institute Pasteur, Paris, France (Pas) [12]. Selected isolates were typed by MLST.

*A. baumannii* strains 28 and 1432 were used as the hosts for phage AS11 and AS12 propagation, respectively. *A. baumannii* strain 28 was isolated in 2002 from a burn patient at the I. I. Dzhanelidze Research Institute of Emergency Medicine, Saint Petersburg, Russia (GenBank accession number MAFT00000000). *A. baumannii* strain 1432 was obtained from the State collection of pathogenic microorganisms and cellular cultures SCPM-Obolensk (accession number B-7134). Both strains were resistant to diverse groups of antibiotics including aminoglycosides, fluoroquinolones, third-generation cephalosporins, and, in the case of *A. baumannii* 1432, carbapenems (imipenem and meropenem).

All bacteria were grown in Luria–Bertani (LB) broth (Difco, Detroit, MI, USA) or Nutrient agar (Himedia Laboratories Pvt. Limited, Mumbai, India) at 37 °C.

### 2.2. Phage Isolation, Propagation and Purification

Clinical materials (burn wound samples) from the I. I. Dzhanelidze Research Institute of Emergency Medicine were used for phage isolation. Non-liquid samples were kept in 0.1 M Tris-HCl buffer, pH 7.0 for 2 h and then cleared by low-speed centrifugation (7000 G for 30 min). The supernatants were incubated for 16–18 h in the presence of growing clinical *A. baumannii* strains of different genotypes at 37 °C, after that chloroform was added. Bacterial debris was pelleted by centrifugation at 7000 G for 30 min followed by filtration of the supernatants through 1.20- and 0.45-µm-pore-size membrane filters, Millex-GV (Millipore, Cork, Ireland). The spot test method as well as plaque assay [13] was used to screen for the presence of lytic phage activity in the resultant filtrates. The plates were incubated overnight at 37 °C and examined for zones of lysis or plaque formation. Single plaques formed on the lawns of sensitive *A. baumannii* strains were picked up in the SM buffer (10 mM Tris-HCl, pH 7.5, 10 mM MgSO_4_, and 100 mM NaCl) and replated three times in order to obtain pure phage stock. 

Phage Fri1 was isolated from fishpond waste near Fribourg in Switzerland.

The phages were propagated using liquid culture of *A. baumannii* host strains (OD_600_ of 0.3) at the multiplicity of infection (MOI) of 0.1. The incubation was performed at 37 °C until complete lysis, and then chloroform was added. Bacterial debris was pelleted by centrifugation at 7000 G for 30 min. The phage lysates were precipitated with polyethylene glycol 6000 (10%, wt/vol)-1 M NaCl at 4 °C overnight, centrifuged at 11000 G for 10 min at 4 °C, re-suspended in the SM buffer, and then spun at 13000 G. The supernatants were treated with DNase (1 µg/mL) and RNase (1 µg/mL) at 37 °C. The nucleases were removed with chloroform. The phage preparations were purified by cesium chloride equilibrium gradient centrifugation at 100000 G (Beckman SW41 Ti rotor, Beckman Coulter Inc., Brea, CA, USA) for 24 h. [14].

### 2.3. Phage Host Range Determination

The lytic activity and host specificity of phages AS11, AS12, and Fri1 were tested against 100 identified genotype-varying MDR *A. baumannii* strains using the double-layer method [13]. For this method, 300 μL of *A. baumannii* bacterial cultures grown in LB medium at 37 °C to OD_600_ of 0.3 was mixed with 4 mL of soft agar (LB broth supplemented with 0.6% agarose). The mixture was plated onto the nutrient agar. Then, the phage suspensions (~10^9^ plaque forming units (PFU) per mL), and their several dilutions were spotted on the soft agar lawns and incubated at 37 °C for 18–24 h.

### 2.4. Phage Adsorption and One-Step Growth Experiments

For adsorption assay, exponentially grown *A. baumannii* cells were mixed with the phages (MOI = 0.001) and incubated at room temperature. A volume of 100 µL of samples was taken in 1, 2, 3, 4, 5, 8, 10, and 15 min and then mixed with 850 µL of SM buffer supplemented with 50 µL of chloroform. After centrifugation, the supernatants were titrated for further determination of unabsorbed phages by the plaque assay method [13] at different time intervals. The adsorption constant was calculated according to the study by Adams [13] for a period of 5 min.

For the one-step growth experiments, 20 mL of host bacterial cells (OD_600nm_ of 0.3) was harvested by centrifugation (7000 G, 5 min, 4 °C) and re-suspended in 0.5 mL LB broth. Bacterial cells were infected with the phage at MOI of 0.01. The bacteriophage was allowed to adsorb for 2 min at 37 °C. Then, the mixture was centrifuged at 10000 G for 2 min to remove unabsorbed phage particles, and the pellet was re-suspended in 20 mL of LB broth. Samples were taken at 5-min intervals over the course of 2 h incubation at 37 °C and immediately titrated.

The procedures were repeated three times.

### 2.5. Electron Microscopy

The phage was examined by negative contrast electron microscopy with procedure [15]. Briefly, the purified and concentrated virus preparations (10^11^ PFU/mL) fixed with 1% glutaraldehyde in 0.1 M phosphate buffer (pH 7.0) were placed on transmission electron microscopy (TEM) support grids followed by rinsing with distilled water several times. The phage samples were stained with 1% uranyl acetate aqueous solution (pH 4.0) for further examination with a Hitachi H-300TM electron microscope (Hitachi Ltd, Tokyo, Japan). The electron microscope magnification was calibrated using the T4 phage as size standard. At least 20 electronic phage images were used for the phage morphology determination.

### 2.6. DNA Isolation and Sequencing

Phage DNA were isolated from concentrated and purified high titer phage stocks by incubation in 0.5% SDS, 20 mM EDTA and 50 µg/mL proteinase K at 56 °C for 1–3 h. The DNA were extracted with phenol–chloroform and then precipitated with ethanol supplemented with sodium acetate [14].

Genome sequencing was performed using Ion Torrent^™^ PGM equipment (Life Technologies, Carlsbad, CA, USA). The generated reads were assembled de novo into single contig using Newbler 2.9 (454 Life Sciences, Branford, CT, USA). Further, the resulting sequences were checked by mapping reads against the assemblies with DNASTAR's Lasergene sequence analysis software version 11.1.0 (DNASTAR Inc., Madison, WI, USA) [16].

Direct terminal repeats were determined as regions of greater coverage of sequencing reads mapped on assembled viral genome contigs. The ends were next verified directly by Sanger sequencing with outward-directed primers located inside and outside putative repeats.

### 2.7. Genome Analysis

Potential open reading frames (ORFs) were identified with the RAST automated annotation engine [17]. Predicted proteins were searched against the NR (non-redundant) database at the NCBI. Additionally, protein sequences were analysed using a Conserved Domain Database (CDD) search at the NCBI [18] and HHpred profile–profile search [19] in order to identify remote sequence similarities. The TMHMM program [20] was used to predict transmembrane helicases. Rho-independent transcriptional terminators were predicted using Arnold [21]. Overrepresented intragenic motifs were found using the MEME program [22]. The RNA polymerase sequences were aligned using Clustal Omega [23]. Phylogenetic trees were constructed by the neighbor-joining method and visualized with FigTree [24]. Comparative analysis of DNA genome sequences was performed using the BRIG software [25]. The presence of tRNAs in the genome sequence was determined using tRNAscan-SE version 1.21 (Santa Cruz, CA, USA) [26].

### 2.8. RNA Purification

*A. baumannii* 28 and 1432 cells were grown to OD_600_ of 0.45 and infected at an MOI of 10 with phages AS11 and AS12, respectively. Infection was stopped at 5-min intervals by rapid chilling. A 0-min time point stands for total RNA from *A. baumannii* 28 and 1432 cells that were not infected with the phages. Cells were collected by centrifugation at 5000 G for 15 min and used for total RNA purification with ExtractRNA (Evrogen JSC, Moscow, Russia). 

### 2.9. Primer Extension and Manual DNA Sequencing

For primer extension reactions, 2 μg of total RNA were reverse-transcribed with 200 U of Maxima Reverse Transcriptase (Thermo Fisher Scientific, Vilnius, Lithuania) according to the manufacturer’s protocol in the presence of 1 pmol of [γ-^32^P] end-labelled gene-specific primers. Reaction products were resolved on 6% sequencing gels. PCR fragments corresponding to the predicted transcription start area were synthesized from AS11 and AS12 DNA. The products of the sequencing reactions, performed with the same end-labelled primers and appropriate PCR fragments as a template using Thermo Sequenase Cycle Sequencing Kit (Affymetrix Inc., Cleveland, OH, USA), were run alongside primer extension reactions. Gels were visualized using Typhoon FLA 9500 (GE Healthcare Bio-Sciences AB, Uppsala, Sweden).

### 2.10. Nucleotide Sequence Accession Numbers

The genome sequences of vB_AbaP_AS11 and vB_AbaP_AS12 phages were submitted to GeneBank. The assigned accession numbers are KY268296 for AS11 and KY268295 for AS12.

## 3. Results and Discussion

### 3.1. Phage Morphological Characteristics, Spectra of Lytic Activity and Infection Parameters

**Figure 1:**
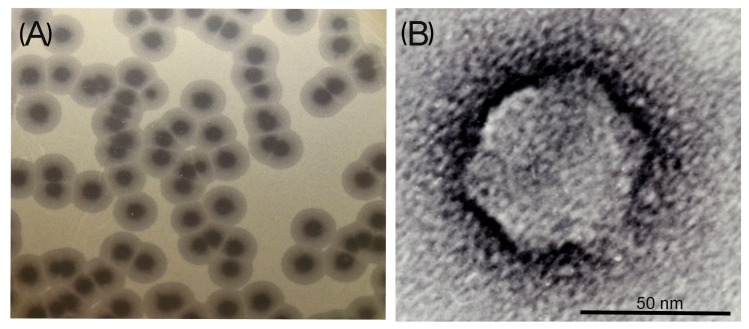
Morphological characteristics of phage AS11: (**A**) Phage plaques with opaque haloes on the *Acinetobacter baumannii* 28 (**B**) Transmission electron micrographs of the bacteriophage. Staining with 1% uranyl acetate. The scale bar is 50 nm.

On sensitive *A. baumannii* strain lawns, AS11 and AS12 form large, clear plaques surrounded by haloes (Figure 1A) which expand in size after overnight incubation at 37 °C, and during subsequent plate storage at 4 °C, while lysis zones of clear plaques remain constant. 

Similar haloes were observed for most described *A. baumannii* phages [6,8,27,28], as well as for some phages infecting other bacterial species [29,30,31,32,33]. The haloes indicate the presence of structural depolymerases in phage virions that degrade external polysaccharide layers of bacterial cells and facilitate the initial stage of phage bacterial host recognition and adsorption [29,31,33].

Phage particle morphology was examined by TEM. AS11 and AS12 were found to have icosahedral heads of 60 nm in diameter and short tails of 10 nm in length (Figure 1B). Thus, the bacterial viruses were classified as morphotype C1 of the family *Podoviridae*. The phages were morphologically similar to earlier isolated and described *A. baumannii* podophages phiAB1, phiAB2 [6], phiAB6 [8], Abp1 [7], and Acibel007 [5].

A total of 100 non-duplicate (one per patient) nosocomial *A. baumannii* isolates, selected to represent diverse geographical origins (53 hospitals of 32 cities of Russia and Belarus), were used to assess the spectra of lytic activity of new phages and phage Fri1. By SNP-typing, the isolates were assigned to 22 genotypes, of which six and five related genotypes corresponded, respectively, to the two dominant international clonal lineages (ICLs) or MLST clonal complexes (CCs): ICL1 (CC109^Oxf^/CC1^Pas^) encompassing 35 isolates and ICL2 (CC92^Oxf^/CC2^Pas^) encompassing 34 isolates. Other international clones identified among the studied isolates included CC103^Oxf^ (*n* = 3), CC110^Oxf^ (*n* = 6), CC113^Oxf^ (*n* = 3), CC147^Oxf^ (*n* = 1), CC253^Oxf^ (*n* = 4), and CC741^Oxf^ (*n* = 1).

An apparent correlation was found between the sensitivity to phages and the genotypes of isolates. In particular, AS12 infected and lysed only representatives of CC110^Oxf^/CC25^Pas^ (six isolates collected from four Russian cities), while AS11 infected isolates of CC253^Oxf^/ST111-ST828^Pas^ (four isolates collected in two Russian cities). 

It is interesting to note that the host range of phage AS11 isolated from a clinical specimen in Russia coincided with that of *A. baumannii* podophage Fri1 isolated from fishpond waste in Switzerland. The coincidence of phenotypic characteristics is most likely caused by the presence of almost identical receptor–recognition proteins or tail spikes with depolymerase activity (gp45 and gp49 for AS11 and Fri1, respectively).

**Figure 2:**
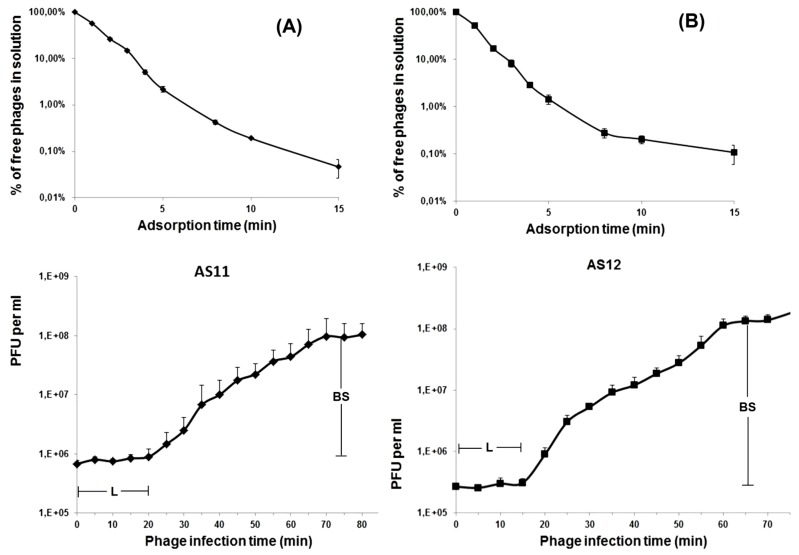
Infection analysis of phages AS11 and AS12. Adsorption assay and one-step growth curve of phage AS11 on *A. baumannii* strain 28 (**A**) and phage AS12 on *A. baumannii* strain 1432 (**B**) with indication of estimated burst size (BS) and latent period (L). Results are the means and standard deviations from three independent experiments. PFU: plaque forming units.

The infection process for both phages was investigated by estimating the AS11 and AS12 adsorption efficiency and one-step growth experiments. As shown in Figure 2, more than 95% of AS11 and AS12 phage particles adsorbed to host cells within 5 min. 

Based on the comparison of adsorption constants (*k*), AS11 (*k* = 4.04 × 10^-8^ mL/min at 5 min) appears to adsorb slightly more slowly than AS12 (*k* = 6.39 × 10^-8^ mL/min at 5 min). The latent periods for AS11 and AS12 were 20 and 15 min, and the burst sizes were approximately 150 and 300 particles per infected cell, respectively. These values are comparable with those reported for other Fri1-like phages phiAB2 [6], Abp1 [7], and *A. baumannii* podovirus vB_AbaP_Acibel007 [5].

### 3.2. AS11 and AS12 Genome Organisation and Comparative Genomic Analysis 

The assembled AS11 and AS12 linear genomic sequences are 41,642 and 41,402 base pairs (bp) long, respectively. AS11 and AS12 genomes share 86% nucleotide sequence identity. Direct terminal repeats (DTRs) of 340 bp for AS11 and 338 bp for AS12 were identified at the genomes’ ends. 

The G+C content of the AS11 and AS12 genomes is 39.29% and 39.31%, respectively, similar to other *A. baumannii* podoviruses and close to the approximate average values for different *A.baumannii* strains (38.94–39.4% according to [34]).

A total of 51 and 49 putative open reading frames (ORFs) were predicted in the AS11 and AS12 genomes, respectively. All of them were transcribed in one (left to right) direction and start with ATG as initiation codon (the only exception is the gene encoding holin in AS11 genome, which starts with TTG). Putative functions were assigned to 29 (14.79%) and 28 (13.72%) products of predicted AS11 and AS12 ORFs, respectively. No tRNA genes were identified.

Almost all of the predicted proteins encoded by AS11 and AS12 have very close homologues in the Fri1 and vB_AbaP_IME200 genomes (Table S1). BLASTp searches indicated that AS11 and AS12 also share a number of homologous genes with LUZ19 [35] (Table S2) and other phiKMV-like phages, including single-subunit viral RNA polymerases (RNAPs) genes. These phages use the bacterial host RNAP for transcription of early phage genes and a phage-encoded RNAP for transcription of middle and late genes during the infection [36,37].

Based on the functions of homologous proteins of AS11 and AS12, likely early, middle and late gene regions can be identified in the AS11 and AS12 genomes.

#### 3.2.1. Early Genome Regions

**Figure 3:**
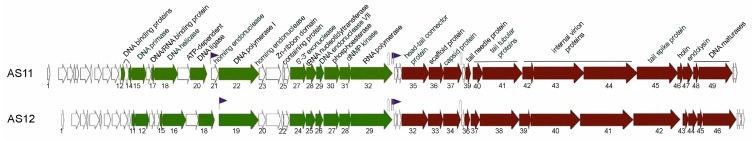
Comparison of AS11 and AS12 genomes. The AS11 and AS12 genomes are schematically presented. Open reading frames (ORFs) are indicated as arrows, the direction of an arrow shows the direction of transcription. The ORFs are colored according to functional predictions: green for genes coding enzymes of nucleotide metabolism and proteins involved in DNA replication and repair; red for virion and lysis protein genes. Overrepresented motifs that likely function as putative phage-encoded RNAP promoters are indicated as violet flags. Terminators are shown as hairpins.

Products of early genes are likely involved in “host takeover” mechanisms at the start of an infection by binding to components of functionally important host systems, inhibiting them or redirecting their activities to serve the needs of the virus [36]. The putative early regions consist of the first fourteen genes of the AS11 phage and the first eleven genes of AS12 (Figure 3, Table S1).

No functions or conserved domains for products of any of these genes could be predicted. The presence of transmembrane helices was predicted inside homologous ORF4 products for both phages.

#### 3.2.2. Middle Genome Regions

Putative middle regions of AS11 and AS12 comprise nucleotide metabolism and DNA replication and repair gene clusters. Genes from these regions encode a DNA primase, DNA helicase, ATP-dependent ligase, DNA polymerase, 5'-3' exonuclease, tRNA nucleotidyltransferase, endonuclease VII, phosphoesterase, deoxy-nucleotide monophosphate (dNMP) kinase, and RNAP (Figure 3, Table S1). ORFs with predicted functions are arranged in the same order for both bacteriophages. The presence of ORFs encoding putative HNH endonucleases has been revealed in these clusters, a common feature for Fri-like phages. In particular, homing endonuclease genes were previously identified in phiAB1 (*gp17*, *gp19*, *gp21*, and *gp24*) [38], phiAB6 (*gp12*, *gp20*, and *gp23*) [8], Abp1 (*gp24* and *gp26*) [7], and Fri1 (*gp25*). In AS12, a homing endonuclease gene is found downstream of the DNA polymerase gene (AS12_*g20*); the AS11 DNA polymerase gene is surrounded by two endonuclease genes (AS11_*g21* and AS11_*g23*). HNH endonucleases located downstream of AS11 and AS12 DNA polymerase genes share 96% identity at the amino acid level. The other AS11 endonuclease, *gp21*, shares only 44% of identity with *gp23* of AS12, but is identical to endonuclease *gp17* of phiAB1. It is interesting that HNH endonuclease genes identified in the genomes of phages Fri1 (*gp25*), phiAB1 (*gp19*), and Abp1 (*gp24*) interrupt the DNA polymerase gene in the same location and probably were integrated into this position in the ancestral phages. 

#### 3.2.3. Late Genome Regions

Late genome regions comprise genes encoding structural proteins, proteins associated with bacterial cell lysis, and with DNA packaging (Figure 3, Table S1). A common set of podovirus structural proteins was predicted including a head-tail connector protein; a scaffolding protein; a major capsid protein; tail tubular proteins A and B; internal virion proteins A, B, and C; and a tail spike. All the predicted proteins except tail spikes are very similar to each other in both phages, and to homologous proteins of other *A. baumannii* podoviruses. Putative tail spikes with predicted exopolysaccharide (EPS) depolymerase domain were found to differ significantly for all *A. baumannii* podophages deposited to public databases, except for AS11 and Fri1 (96% identity at amino acid level). The host lysis gene clusters of AS11 and AS12 comprise endolysins (*gp44* and *gp47*, respectively) and holin proteins. The coding regions of corresponding genes overlap. According to Young et al. [39], the holins belong to class I based on the presence of three transmembrane domains, with “N-out, C-in” topology. The Rz–Rz1 proteins identified in the lysis cassette of *Pseudomonas* phiKMV-like phages [36] are absent from the genomes of AS11 and AS12 and related *A. baumannii* phages.

#### 3.2.4. Comparative Genomics

At the moment several complete genome sequences of *A. baumannii* infecting podophages (phages Fri1, vB_AbaM_IME200, Abp1, phiAB6, phiAB1, vB_AbaP_PD-AB9, vB_AbaP_PD-6A3, WCHABP5, Petty, vB_AbaP_Acibel007) and several partial genome sequences (phages AB3, phiAB2, vB_AbaP_CEB1, vB_AbaP_CEB2, ABP-01, and ABP-04) are available in the GenBank database. 

**Figure 4:**
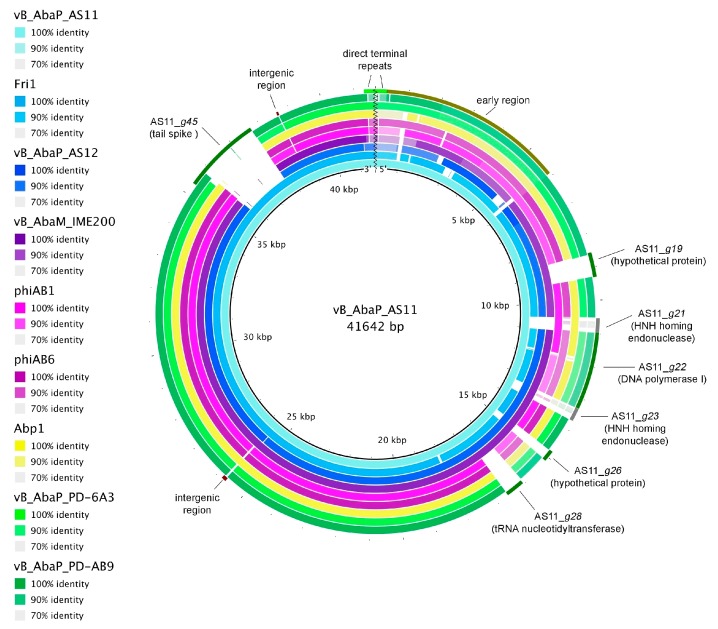
Comparative genome analysis of all known to date Fri1-like viruses. Reference: *A. baumannii* phage vB_AbaP_AS11 genome. Query: complete genome sequences of related *A. baumannii* podoviruses, listed in the key left. The DNA regions of AS11 genome that differ from other phages are marked. Map includes divider line showing genomes are linear.

A comparison of all known *A. baumannii* podophages revealed high similarity on the DNA level between phages AS11, AS12, Fri1, vB_AbaM_IME200, Abp1, phiAB1, phiAB6, vB_AbaP_PD-AB9, and vB_AbaP_PD-6A3. The most variable genome regions of all the described Fri1-like phages fall in several early genes, genes encoding some hypothetical proteins and tRNA nucleotidyltransferase in the middle genome regions, areas surrounding the DNA polymerase I gene with integrated HNH homing endonucleases, some intergenic regions, and regions of host receptor-binding–recognition genes (tail spikes) (Figure 4).

The diversity of tail spikes provides phages with the ability to specifically infect only certain bacterial strains that have cognate receptors on the bacterial surfaces.

**Figure 5:**
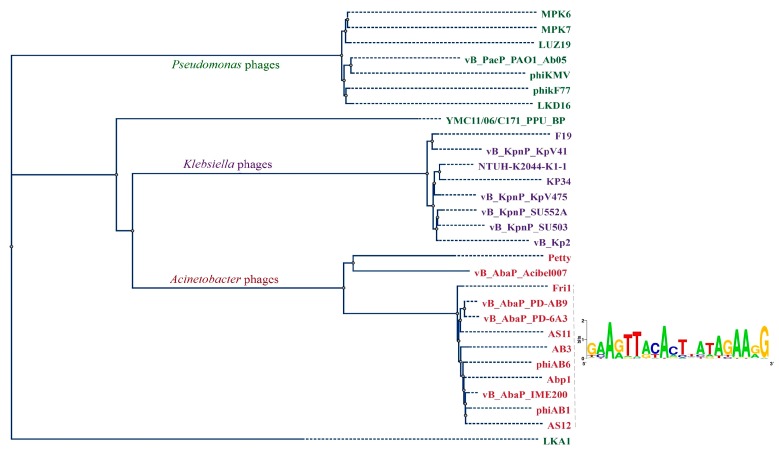
Comparison of RNA polymerases (RNAP) proteins. Phylogenetic tree of single-subunit RNAPs from Fri1-like, phiKMV-like, and Kp34-like phages constructed using a neighbor-joining algorithm is shown. Consensus sequences for overrepresented motifs from intergenic regions of indicated *A. baumannii* phages are shown.

To confirm the clustering of AS11 and AS12 within the Fri1-like phages group among *A. baumannii* podophages and other viruses with similar genome organization, phylogenetic analysis of their RNA (Figure 5) and DNA polymerase proteins (Figure S1) was performed. 

The RNA polymerase-based phylogenetic tree revealed that all *A. baumannii* podoviruses belonging to *Autographivirinae* subfamily, as expected, form a distinct clade which can be subdivided into two groups (Figure 5). The first group comprises phages AS11, AS12, Fri1, vB_AbaM_IME200, Abp1, phiAB1, phiAB6, vB_AbaP_PD-AB9, vB_AbaP_PD-6A3, and the partially sequenced phage AB3, which share a high percentage of DNA similarity according to comparative genome analysis. Consensus sequences for overrepresented motifs from intergenic regions of these phages are also presented in Figure 5. The second group comprises phages vB_AbaP_Acibel007 and Petty that seem to be more evolutionarily distant from the *A. baumannii* phages of *Fri1virus*, but nevertheless they possess the same genomic architecture. 

Phages AS11 and AS12 also share similar genome organisation with phiKMV-like viruses [35,36,37], but some exceptions can be noted: maturases A and B of *A. baumannii* phages are located not in front but after genes responsible for lysis “from within”. One more distinctive feature of viruses infecting *A. baumannii* is the presence of genes with the predicted function of dNMP kinase in front of RNAP genes.

### 3.3. Regulation of Transcription

Based on overall DNA and protein sequence identity and phylogeny, phages similar to *Fri1virus* are considered as a separate group within *Autographivirinae* subfamily of *Podoviridae* family [9]. Nevertheless, they evidently share similar genome organization and a number of homologous genes with phiKMV-like viruses belonging to the T7-supergroup, which are known to use two types of RNAP for transcription of their own genes during development: (i) host RNAP for transcription of early genes from σ70-dependent promoters, and (ii) phage-encoded single-subunit RNAP for transcription of other genes.

Viral RNAPs of T7-like phages transcribe the middle gene encoding proteins involved in phage DNA replication and late genes encoding phage structural proteins [36,37]. The mechanisms for the prevention of mutual influence of host and viral RNAPs have been described for some representatives of the T7-supergroup. In particular, the product of the phage T7 middle gene 2 inhibits host RNAP [40] by binding to its β’ subunit and preventing open promoter complex formation by the host σ^70^ RNAP holoenzyme [41]. Host RNAP inhibitors encoded by phiKMV-like phages infecting *Pseudomonas* have also been identified. These proteins share a common RNAP binding site with T7 gp2 and can functionally substitute for it [41]. Considering the similarity of genome organization, it can be assumed that *A. baumannii* Fri1-like podophages most likely encode gp2-like proteins affecting host RNAP. In the genomes of *Pseudomonas* phiKMV-like viruses, the inhibitors are encoded by genes located immediately upstream of respective viral RNAP genes: LUZ19_*g*25.1, PT2 and PT5_*g*25.1, LKD16_*g*25.b, and LKA1_*g*36 [42]. However, as mentioned above, AS11 and AS12 genomes contain genes with the predicted function of dNMP kinase directly in front of RNAP sequences (Figure 3). In fact, no gp2-like homologs were detected in available annotations of *A. baumannii* phage genomes. There are only two predicted ORFs with unknown functions located between the genes encoding RNAPs and head-tail connector proteins in AS11 and AS12 structural genome clusters (Figure 3). No similarity of the products of these ORFs with gp2 homologs of T7 supergroup phages was revealed. Therefore, it will subsequently be a subject of interest to detect the AS11 and AS12 proteins binding to host RNAP using additional approaches such as co-affinity purification coupled with mass spectrometry [43].

Promoters for host RNAP are localized at the left end of the T7 genome that is injected first, promoters for phage-encoded RNAP can be found throughout the T7 genome after the RNAP gene [44]. It was shown for phiKMV-like phages that the localization of promoters is similar to T7 [35,36]. 

We predicted putative promoters in the AS11 and AS12 genomes by searching for overrepresented sequence motifs in all intergenic regions using the MEME program [19]. This search resulted in three matches for each phage. Predicted promoters display a rightward orientation like all genes annotated in the phage genomes. First promoters for both phages are located in the intergenic region upstream of the gene 19 encoding DNA polymerase I in the AS12 genome and the gene 21 encoding HNH homing endonuclease prior to the DNA polymerase I in the AS11 genome. Two additional closely located putative promoters were predicted in intergenic regions after genes 32 and 29 that encode T7 RNAP for AS11 and AS12, respectively (Figure 3). The location of predicted promoters and two predicted transcription terminators, which are located after the RNA polymerase gene (AS11_*gp*32) and major capsid protein (AS11_*gp*37), confirms the clustering of genes (Table S3).

**Figure 6:**
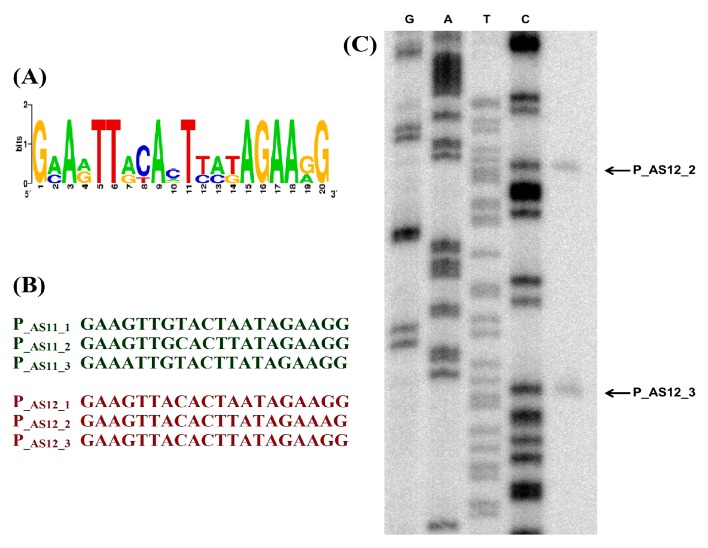
Identification of AS11 and AS12 phage-encoded T7-type RNAP promoters: (**A**) A logo representation of putative promoter consensus sequence for phages AS11 and AS12; (**B**) Sequences of phage-encoded promoters validated by primer extension analysis; (**C**) Example of in vitro promoter mapping. The products of sequencing reactions, performed with the same end-labelled primers and appropriate PCR fragment as a template were run alongside primer extension reactions.

Bioinformatically predicted phage-encoded promoters were confirmed by in vivo transcription analysis. Cells were infected with phages AS11 and AS12. Total RNA collected at 5 min points of infection was analyzed with [γ-32P] end-labelled primers annealing to corresponding coding regions. To exactly identify the transcriptional start sites, DNA sequencing reactions with corresponding PCR-amplified AS11 and AS12 genome fragments and primers used for the primer extension reaction were performed (Figure 6).

### 3.4. Phage Host Recognition Proteins and Primary A. baumannii Bacterial Receptors

Phages AS11 and AS12 seem to be promising for medical use as they infect and lyse clinically important MDR *A. baumannii* strains, but susceptible bacterial isolates constitute a small portion of our collection. This situation is common for other *A. baumannii* podoviruses [6,7,8].

As mentioned above, comparative genome analysis revealed fairly high DNA sequence similarity between all Fri1-like podophages, but no homology in regions encoding C-terminal domains of tail spikes carrying EPS degrading activity, except phages AS11 and Fri1 (Figure 4). The N-terminal protein domains responsible for attachment to the phage particles are very conserved within the tail spikes of presented in the database *A. baumannii* podoviruses. HHpred data predicts that the tail spikes are trimeric beta-structural glycoside hydrolases of family 28. The C-terminal domain structure of AS12 phage tail spikes has been predicted as intramolecular chaperone. Similar structures are located at the C-terminus of phage fibers and tail spikes, for example, in *E. coli* virus T5 (PDB ID: 4UW8), *Bacillus* virus GA1 (PDB ID: 3GUD), and *E. coli* phage K1F (PDB ID: 3GW6).

Deletion mutants lacking N-terminal domains of AS11, Fri1 and AS12 tail spikes (gp45, gp49, and gp42, respectively) were cloned, expressed and purified (data will be published elsewhere). Purified depolymerases are highly specific and form opaque haloes on lawns of *A. baumannii* strains, which are sensitive to phages that encode them (Figure S2). The structural depolymerases are responsible for the cleavage of capsular polysaccharide (CPS) layers during phage attachment to the host cell [29,31,33]. Thus, CPS can be suggested as the primary receptors for depolymerase-carrying *A. baumannii* phages.

The structure of the N-terminally-truncated tail spike and corresponding CPS depolymerisation products of phage phiAB6 infecting *A. baumannii* have been published recently [45]. The tail spike structure revealed a trimeric *β*-helix architecture that bears intersubunit carbohydrate-binding grooves identified as the determinant of product specificity. Taking into account these results and our data on the determination of phage and purified depolymerase activities on a representative collection of *A. baumannii* strains, it can be assumed that all *A. baumannii* strains sensitive to phages with particular tail spikes are likely producing the same type of CPS. In other words, the variety of tail spikes structures most likely determines the host range specificity. Experimental confirmation of this statement could be found in other recent work [8], in which the replacement of tail spikes in *A. baumannii* podophages phiAB1 and phiAB6 (ORF41 and ORF40, respectively) and the construction of the chimeric phage phiAB1 carrying the tail spike protein of phiAB6 (ORF40) instead of phiAB1 (ORF41) has led to a change of host specificity.

The nuclear magnetic resonance structure of the CPS of *A. baumannii* strains 28 and 1432, bacterial hosts for phages AS11 (the same for phage Fri1) and AS12, respectively, have been already determined. The capsule biosynthesis gene clusters containing the genes responsible for the synthesis, assembly, and export of the polysaccharides have been identified and assigned to KL19 and KL27 (KL, chromosomal K locus) for *A. baumannii* strains 28 [46] and 1432 [47], respectively.

By now, more than 100 types of chromosomal capsule locus (KL types) have been identified in *A. baumannii* strains. Thus, we suggest that evolutionary bacteriophages had to adopt an equal variety of specific recognition proteins to different KL types. In this study, we summarized data on the correlation of identified tail spikes encoded in *A. baumannii* podophage genomes to the KL types of their host strains (Table S4). From the available data, it has been concluded that phages with differing tail spike structures are capable of infecting different KL types within *A. baumannii* species. Thus, we propose that the host range breadth of depolymerase-carrying podophages depends on the prevalence and dominance of *A. baumannii* strains with the appropriate capsular polysaccharides structure in a certain environment.

## Supplementary Materials

The following are available online at www.mdpi.com/1999-4915/9/7/188/s1, Figure S1: Comparison of DNA polymerase proteins, Figure S2: Activities of Fri1, AS11, and AS12 recombinant depolymerases after 9 hours incubation at 37 °C, Table S1: General information about predicted AS11 and AS12 gene products, Table S2: AS11 and LUZ19 homologues genes, Table S3: Putative AS11 and AS12 terminators, Table S4. Correlation of podophage tail spikes to KL types of *A. baumannii* host strains.
