# Supplementary material for: Novel Fri1-like Viruses Infecting Acinetobacter baumannii—vB_AbaP_AS11 and vB_AbaP_AS12—Characterization, Comparative Genomic Analysis, and Host-Recognition Strategy
Source: Viruses. 2017 Jul 17;9(7):188. doi: 10.3390/v9070188 (PMC5537680; doi:10.3390/v9070188)
Supplement: Supplementary file 1 [file viruses-09-00188-s001.docx]

**Supplementary Materials**


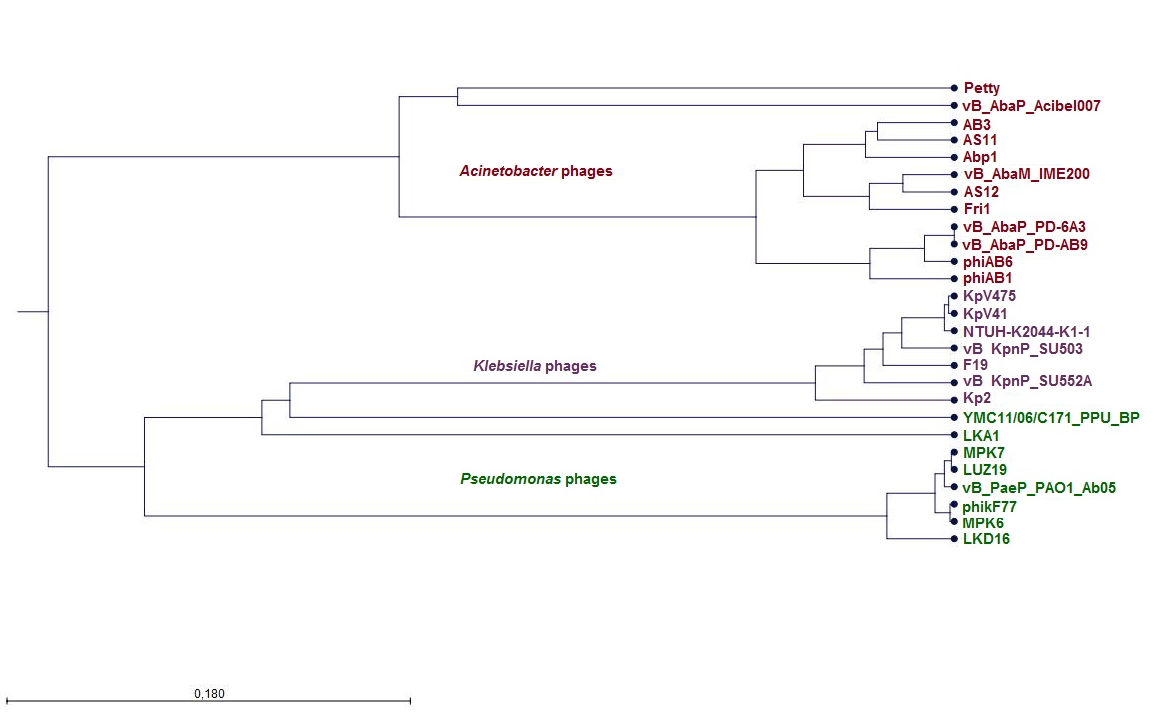


**Figure S1**. Comparison of DNA polymerase proteins. Phylogenetic tree of DNA polymerases from Fri1-like, phiKMV-like, and Kp34-like phages constructed using UPGMA algorithm is shown.

**
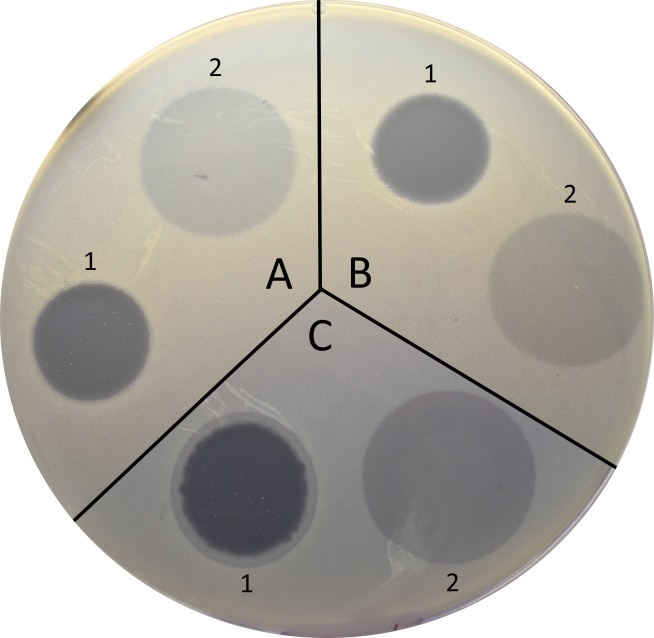
**

**Figure S2.** Activities of Fri1, AS11, and AS12 recombinant depolymerases after 9 hours incubation at 37 °C. (**A**) Spot test of phage Fri1 (1) and its tail spike protein (2) on the bacterial lawn of *A. baumannii* 28 belonging to KL19 (**B**) Spot test of phage AS11 (1) and its tail spike (2) on the bacterial lawn of *A. baumannii* 28 (**C**) Spot test of phage AS12 (1) and its tail spike protein (2) on the bacterial lawn of *A. baumannii* 1432 belonging to KL27. In all spot tests 10 μL of each phage (~109 PFU/mL) and 10 μL of each depolymerase (10–50 µg) were taken.

**Table S1.** General information about predicted AS11 and AS12 gene products. Homologues genes from phages AS11 to AS12 are typed in front of each other.

| **Gene** | **bp** | **Product** | **Domain** | | **Best BLASTp Match for AS11; *E*-value** | | **HHpred Search** | |
| --- | --- | --- | --- | --- | --- | --- | --- | --- |
| *AS11_g01* | 150 | hypothetical protein |  | | - | |  | |
| *AS12_g01* | 159 | hypothetical protein | PF10545 | | - | |  | |
| *AS11_g02; AS12_g02* | 510 | conserved  hypothetical protein | PHA01782 | | hypothetical protein phage Abp1  YP_008058195.1, 5e-119 | |  | |
| *AS11_g03; AS12_g03* | 375 | hypothetical protein | PF13937 | | hypothetical protein phage vB_AbaP_IME200 YP_009216499.1, 2e-75 | |  | |
| *AS11_g04; AS12_g04* | 114 | hypothetical membrane protein |  | | hypothetical protein phage vB_AbaP_IME200 YP_009216500.1, 2e-09 | |  | |
| *AS11_g05* | 225 | hypothetical protein |  | | hypothetical protein phage Fri1  YP_009203013.1, 1e-46 | |  | |
| *AS11_g06; AS12_g05* | 597 | hypothetical protein |  | | hypothetical protein phage vB_AbaP_IME200 YP_009216502.1, 3e-144 | |  | |
| *AS11_g07; AS12_g06* | 114 | hypothetical membrane protein |  | | hypothetical protein phage Fri1  YP_009203015.1 5e-16 | |  | |
| *AS11_g08; AS12_g07* | 399 | hypothetical protein |  | | hypothetical protein phage Fri1  YP_009203017.1 6e-92 | |  | |
| *AS11_g09; AS12_g08* | 495 | hypothetical protein |  | | hypothetical protein phage vB_AbaP_IME200 YP_009216506.1 9e-96 | |  | |
| *AS11_g10; AS12_g09* | 438 | hypothetical protein |  | | hypothetical protein phage vB_AbaP_IME200 YP_009216507.1 4e-97 | |  | |
| *AS11_g11; AS12_g10* | 168 | hypothetical protein | PLN03086 | | hypothetical protein phage vB_AbaP_IME200 YP_009216508.1 7e-30 | |  | |
| *AS11_g12* | 201 | DNA binding protein | zn-finger | | hypothetical protein *Acinetobacter* phage Fri1 YP_009203021.1 2e-16 | | ubiquitin-protein ligase, e-value=0.0018 | |
| *AS11_g13* | 240 | hypothetical protein |  | | hypothetical protein phage phiAB6  ALA12234.1 2e-23 | | carotenogenesis protein CARS, e-value=0.0026 | |
| *AS11_g14; AS12_g11* | 210 | DNA binding protein | zn-finger | | hypothetical protein phage Abp1  YP_008058207.1 2e-36 | | ubiquitin-protein ligase MIB1, e-value=0.00014 | |
| *AS11_g15; AS12_g12* | 801 | DNA primase | PF13155 | | DNA primase/helicase phage vB_AbaP_IME200 YP_009216512.1 0.0 | |  | |
| *AS12_g13* | 315 | hypothetical protein |  | | hypothetical protein Fri1_18 phage Fri1 YP_009203024.1 4e-15 | |  | |
| *AS11_g16; AS12_g14* | 318 | hypothetical protein |  | | hypothetical protein phage vB_AbaP_IME200 YP_009216513.1 2e-59 | |  | |
| *AS11_g17; AS12_g15* | 246 | DNA/RNA binding protein |  | | hypothetical protein phage Fri1  YP_009203026.1 3e-49 | | sigma factor, e-value=1.6; sigma-70 region 2,  e-value=0.22 | |
| *AS11_g18; AS12_g16* | 1299 | DNA helicase |  | | putative DNA helicase phage phiAB1  YP_009189354.1 0.0 | |  | |
| *AS11_g19; AS12_g17* | 738 | hypothetical protein | PF11296 | | hypothetical protein phage vB_AbaP_IME200 YP_009216516.1 3e-172 | |  | |
| *AS11_g20; AS12_g18* | 978 | ATP-dependent  DNA ligase | PHA00454 | | putative ATP-dependent DNA ligase phage Fri1 YP_009203029 0.0 | |  | |
| *AS11_g21* | 447 | HNH homing endonuclease | PF13392 | | putative HNH endonuclease phage phiAB1 YP_009189356.1 5e-105 | | HNH homing endonuclease *Bacillus* phage SPO1,  e-value=4.7e-27 | |
| *AS11_g22; AS12_g19* | 2319 | DNA polymerase I | PF00476 PF10542 | | putative DNA polymerase phage AB3  YP_008060158.1 0.0 | |  | |
| *AS11_g23; AS12_g20* | 396 | HNH homing endonuclease | PF13392 | | hypothetical protein phage vB_AbaP_IME200 YP_009216519.1 1e-89 | | HNH homing endonuclease; *Bacillus* phage SPO1,  e-value=4.6e-25 | |
| *AS11_g24; AS12_g21* | 891 | conserved  hypothetical protein | PHA02030 | | hypothetical protein AB3_0023 phage AB3 YP_008060156.1 0.0 | | PF14714, KH-domain-like of EngA bacterial GTPase enzymes, (MotifFinder), e-value=0.056 | |
| *AS11_g25; AS12_g22* | 120 | Zn-ribbon domain containing protein | PRK04000 | | hypothetical protein Abp1_0028 phage Abp1 YP_008058220.1 6e-29 | | zinc binding protein, FHA from *Sulfolobus acidocaldarius*, e-value=1.7e-07 | |
| *AS11_g26; AS12_g23* | 336 | hypothetical protein | PF11072 | | hypothetical protein AB3_0022 phage AB3 YP_008060155.1 8e-76 | | DNA repair/replication protein, ATP- binding domain from *Geobacillus stearothermophilus*,  e-value=0.79 | |
| *AS11_g27; AS12_g24* | 954 | 5'-3' exonuclease | PF02739 | | hypothetical protein phage vB_AbaP_IME200 ANW09791.1 0.0 | | 5'-3' exonuclease (MotifFinder) e-value=0.097; hydrolase-DNA complex, flap endonuclease from *Escherichia coli*, e-value=2.2e-38 | |
| *AS11_g28; AS12_g25* | 567 | tRNA nucleotidyltransferase | COG0617 | | hypothetical protein phage vB_AbaP_IME200 YP_009216524.1 5e-116 | | nucleotidyltransferase domain (MotifFinder),  e-value=0.019;  nucleotidetransferase from *Escherichia coli* | |
| *AS11_g29; AS12_g26* | 441 | DNA endonuclease VII | PF02945 | | putative DNA endonuclease VII phage Abp1 YP_008058223.1 9e-105 | |  | |
| *AS11_g30; AS12_g27* | 936 | phosphoesterase with HTH domain | PF13384 PF00149 | | hypothetical protein Abp1_0032 phage Abp1 YP_008058224.1 0.0 | | C-terminus: Mn2+ binding, hydrolase from *Streptococcus pneumoniae*, e-value=6.3e-16;  N-terminus: helix-turn-helix,  DNA binding protein, e-value=1.6e-05 | |
| *AS11_g31; AS12_g28* | 651 | dNMP kinase | PHA02575 | | hypothetical protein Abp1_0033 phage Abp1 YP_008058225.1 7e-154 | |  | |
| *AS11_g32; AS12_g29* | 2418 | RNA polymerase,  T7-like | PF00940 PF14700 | | putative DNA-directed RNA-polymerase phage Fri1 YP_009203042.1 0.0 | |  | |
| *AS11_g33; AS12_g30* | 198 | hypothetical protein |  | | hypothetical protein phiAB1_gp29 phage phiAB1 YP_009189368.1 1e-40 | |  | |
| *AS11_g34; AS12_g31* | 252 | hypothetical protein |  | | hypothetical protein phage vB_AbaP_IME200 YP_009216530.1 8e-53 | | PF01231, Indoleamine 2,3-dioxygenase (MotifFinder), e-value=0.1 | |
| *AS11_g35; AS12_g32* | 1557 | head-tail connector protein | PF12236 | | collar protein phage vB_AbaP_IME200 YP_009216531.1 0.0 | |  | |
| *AS11_g36; AS12_g33* | 861 | scaffold protein | PHA01929 | | putative scaffolding protein phage vB_AbaP_PD-AB9 YP_009189839.1 0.0 | |  | |
| *AS11_g37; AS12_g34* | 1038 | capsid protein | PHA02004 PHA00658 | | capsid protein phage Fri1  YP_009203047.1 0.0 | |  | |
| *AS11_g38; AS12_g35* | 186 | hypothetical protein |  | | hypothetical protein Fri1_42 phage Fri1 YP_009203048.1 2e-33 | | PF16331, TolA binding protein trimerisation (MotifFinder), e-value=0.01 | |
| *AS11_g39; AS12_g36* | 294 | tail needle protein |  | | hypothetical protein Fri1_43 phage Fri1 YP_009203049.1 5e-56 | | PF06810, phage minor structural protein (MotifFinder) e-value=0.017; tail needle protein from bacteriophage P22, e-value=6.4e-07 | |
| *AS11_g40; AS12_g37* | 561 | tail tubular protein A | PHA00428 | | tail fibers protein phage vB_AbaP_IME200 YP_009216536.1 1e-132 | |  | |
| *AS11_g41; AS12_g38* | 2292 | tail tubular protein B |  | | putative tail tubular protein B phage vB_AbaP_PD-AB9 YP_009189834.1 0.0 | |  | |
| *AS11_g42; AS12_g39* | 672 | internal virion protein A |  | | putative internal virion protein B phage phiAB6 ALA12261.1 3e-159 | |  | |
| *AS11_g43; AS12_g40* | 2886 | internal virion protein B | cd00980 | | structural protein phage vB_AbaP_PD-6A3 YP_009190470.1 0.0 | |  | |
| *AS11_g44; AS12_g41* | 3099 | internal virion protein C | PHA03413 | | hypothetical protein phage vB_AbaP_IME200 YP_009216488.1 0.0 | |  | |
| *AS11_g45* | 2352 | tail spike protein | COG5434 | | tail spike protein phage Fri1  YP_009203055.1 0.0 | | EXO-poly-alpha-D-galacturonosidase from *Thermotoga maritima*, e-value=4.7e-21 | |
| *AS12_g42* | 2706 | tail spike protein | PF03906 PF16535 PF13884 | | hypothetical protein *Acinetobacter gyllenbergii* WP_023270272.1 0.0 | | binds to the polymannose O-antigen accelerating adsorption of T5 phage; peptidase domain | |
| *AS11_g46; AS12_g43* | 336 | holin | PF05106 | | putative holin phage vB_AbaP_PD-AB9 YP_009189829.1 8e-73 | | three transmembrane helixes, N-term signal sequence; phage holin family (MotifFinder),  e-value=1.2e-06 | |
| *AS11_g47; AS12_g44* | 558 | endolysin | chitinase_glyco_hydro_19 | | endolysin *Acinetobacter* phage Fri1  YP_009203057.1 2e-130 | |  | |
| *AS11_g48; AS12_g45* | 309 | DNA maturase A |  | | hypothetical protein Abp1_0051 phage Abp1 YP_008058243.1 4e-66 | | PF03299, Transcription factor  AP-2 (MotifFinder), e-value=0.053 | |
| *AS11_g49; AS12_g46* | 1938 | DNA maturase B | smart00487 | | putative DNA maturase B phage phiAB1 YP_009189384.1 0.0 | |  | |
| *AS11_g50; AS12_g47* | 135 | hypothetical protein |  | | hypothetical protein phage vB_AbaP_IME200 YP_009216494.1 6e-21 | | integration HOST factor beta-subunit from *Escherichia coli*, e-value=2.6 | |
| *AS11_g51; AS12_g48* | 204 | hypothetical protein |  | | hypothetical protein Abp1_0054 phage Abp1 YP_008058246.1 2e-36 | |  | |
| *AS12_g49* | 183 | hypothetical protein |  | - | |  | |  |

**Table S2.** AS11 and LUZ19 homologues genes.

| **LUZ19 gene** | **Max** | **Total** | ***E*-value** | **Identity** | | **AS11 gene** |  |
| --- | --- | --- | --- | --- | --- | --- | --- |
| PPLUZ19_gp19 DNA polymerase | 554 | 580 | 9e-175 | 41% | AS11_gp22 DNA polymerase I | | |
| PPLUZ19_gp43 terminase large subunit | 485 | 485 | 9e-154 | 43% | AS11_gp49 DNA maturase B | | |
| PPLUZ19_gp26 RNA polymerase | 344 | 344 | 1e-102 | 32% | AS11_gp32 RNA polymerase, T7-like | | |
| PPLUZ19_gp30 head-tail connector protein | 288 | 288 | 5e-87 | 38% | AS11_gp35 head-tail connector protein | | |
| PPLUZ19_gp34 tail tubular protein B | 283 | 283 | 6e-82 | 27% | AS11_gp41 tail tubular protein B | | |
| PPLUZ19_gp15 DNA_B Helicase | 202 | 202 | 2e-58 | 33% | AS11_gp18 DNA helicase | | |
| PPLUZ19_gp22 5'-3' exonuclease | 186 | 186 | 7e-54 | 40% | AS11_gp27 5'-3' exonuclease | | |
| PPLUZ19_gp32 major capsid protein | 167 | 167 | 2e-47 | 32% | AS11_gp37 capsid protein | | |
| PPLUZ19_gp23 endonuclease type 7 | 109 | 109 | 2e-29 | 50% | AS11_gp29 DNA endonuclease VII | | |
| PPLUZ19_gp21 hypothetical protein | 113 | 113 | 1e-28 | 37% | AS11_gp24 conserved hypothetical protein | | |
| PPLUZ19_gp14 primase | 99.8 | 99.8 | 1e-24 | 29% | AS11_gp15 DNA primase | | |
| PPLUZ19_gp3 hypothetical protein | 92.8 | 92.8 | 3e-23 | 38% | AS11_gp02 conserved hypothetical protein | | |
| PPLUZ19_gp33 tail tubular protein A | 68.9 | 68.9 | 5e-15 | 26% | AS11_gp40 tail tubular protein A | | |
| PPLUZ19_gp42 terminase small subunit | 39.7 | 39.7 | 1e-05 | 35% | AS11_gp48 DNA maturase A | | |
| PPLUZ19_gp20 hypothetical protein | 33.9 | 33.9 | 0.001 | 40% | AS11_gp26 hypothetical protein | | |

**Table S3.** Putative AS11 and AS12 terminators. Sequence (parenthesis or colors indicate terminator stem). Lowercase letters in RNAmotif predictions indicate the spacer element, between the stem-loop and T-rich region.

| **5' End**  **Position** | **Sequence** | **Free energy of stem-loop region (kcal/mol)** |
| --- | --- | --- |
|  | **AS11** |  |
| 12985  18372  21078  25132  40219 | ACUUCUGUCAAGGGGAAGCAUCCCUUAUUGUUCAAG  AGGACGUGCUAUUGAUCCUAAUAUUUGGGUUAAUUAUCUUAAGAA  GGUUAAACAUGGCCCACAUACUUUCGAGUAUGUGGGCUUUUUUUUUUUU  AAUUAUAACAUGGAGGGCUUAACGGCUCUCCUUUCUAUUUAGG  AACUCCUAUAUUUACUUACUAUCGGUAGGUGGUUUUAAGGGUGG | -3.70  -6.20  -12.70  -12.60  -6.40 |
|  | **AS12** |  |
| 17622  20329  20337  24366  25695  36779  39799 | AGGGCGUGCUAUUGAUCCUGAUAUUUGGGUUAAUUAUCUUAAGAA  GUUAAUACUAAGCCCAUGUGCCUUGAGCAUGUGGGCUUUUUUUUUUUU  UAAGCCCAUGUGCCUUGAGCAUGUGGGCUUUUUUUUUUUU  AAUAAUAACAUGGAGGGCUUAACGGCUCUCCUUUCUAUUUAGG  UAUACGCUUGAUUGACAUCAACGGUGUUAAUUAUAUUAUGAU  CACGUAUGAUCUAGGCUCAUCAGGGUCUAcUUUUAGAAACGC  AACUCUUAUAUUUACUUACUAUCGGUAGGUGGUUUUAAGGGUGG | -6.20 -10.80 -4.40 -12.60  -5.10  -7.00  -6.40 |

**Table S4.** Correlation of podophage tail spikes to KL types of *A. baumannii* host strains

| ***A. baumannii***  **Podophages** | **Phage Genome Accession Number** | **Accession Number of**  **Tail Spike Protein** | ***A. baumannii***  **Host Strain** | **KL Type of**  **Host Strain** |
| --- | --- | --- | --- | --- |
| Fri1 | NC_028848.1 | YP_009203055.1 | 28 | KL19 [45] |
| vB_AbaP_AS11 | KY268296 | AQN32697.1 | 28 | KL19 [45] |
| vB_AbaP_AS12 | KY268295 | APW79830.1 | 1432 | KL27 [46] |
| phiAB1 | NC_028675.1 | YP_009189380.1 | M68316 [8] | na* |
| phiAB6 | NC_031086.1 | YP_009288671.1 | 54149 [8] | KL2 [44] |
| Abp1 | NC_021316.1 | YP_008058239.1 | AB1 [7] | na |
| vB_AbaM_IME200 | NC_028987.1 | YP_009216489.1 | na | na |
| vB_AbaP_PD-AB9 | NC_028679.1 | YP_009189830.1 | na | na |
| vB_AbaP_PD-6A3 | NC_028684.1 | YP_009190472.1 | na | na |
| WCHABP5 | KY888680.1 | ARQ94869.1 | WCHAB1334 | KL2 |
| AB3 (partial) | NC_021337.1 | YP_008060136.1 | na | na |
| phiAB2 (partial) | GU979517.1 | GU979517.1 | M3237 [6] | KL3 |
| vB_AbaP_Acibel007 | NC_025457.1 | YP_009103257.1 | 070517 [5] | KL1 |

* not available
